# Psychological distress during the COVID-19 pandemic: An integrative perspective

**DOI:** 10.1371/journal.pone.0293189

**Published:** 2023-10-26

**Authors:** Michal Mahat-Shamir, Ester Zychlinski, Maya Kagan

**Affiliations:** School of Social Work, Ariel University, Ariel, Israel; St John’s University, UNITED STATES

## Abstract

Informed by socio-ecological psychology and the conservation of resources model, the present study proposes an integrative perspective on the association between psychological distress and a constellation of factors, during the COVID-19 outbreak in Israel. Our sample, comprised of 991 adult participants, was measured for psychological distress, locus of control (internal/ external), resilience, loneliness, social support, dimensions of citizens’ trust in government organizations (perceived competence, benevolence, and integrity), and demographic characteristics. The findings showed that women, non-religious people, and the unemployed reported higher levels of psychological distress. Internal locus of control, resilience, social support, and the extent to which citizens perceive government organizations as benevolent were negatively associated with psychological distress. Self-reported loneliness and external locus of control positively predicted the level of respondent psychological distress. No association was detected between age, competence and integrity and psychological distress. An overview of the research findings indicates that individuals with greater resources were less likely to suffer from psychological distress during the COVID-19 outbreak. These findings call upon mental health care practitioners to help as well as to enable clients to attain resources in order to lower their levels of psychological distress. Policies developed by policymakers during periods of acute crisis should consider the specific needs and vulnerabilities of certain population groups, including women and the unemployed who may be more susceptible to psychological distress. It is also important for policymakers to be aware that the perception of democratic governments as benevolent can serve as a buffer against psychological distress during times of crisis.

## Introduction

COVID-19 has been the cause of significant morbidity worldwide. By the end of March 2021 (the period in which questionnaires were distributed for this study), there were more than 18,300 active cases and 6,085 deaths attributed to COVID-19 in Israel [1]. The physical threat posed by the pandemic was accompanied by major psychological concerns due to insecurity and isolation caused by the COVID-19 crisis and the measures used to mitigate virus transmission [2–5]. These stress-inflicting components have been found to negatively affect individuals’ mental health and to increase levels of psychological distress [6]. Nevertheless, as the literature highlights the multifactorial nature of individual differences in both reactions and adaptation to stress, the current study’s aim was to examine the factors contributing to psychological distress during the COVID-19 pandemic. Socioecological psychology [7] and stress and coping resource theories [8] provide valuable theoretical perspectives germane to this exploration. Socio-ecological psychology’s fundamental premise is that our thoughts, feelings, and behavior are mutually affected by our ecologies (namely, socioecological environments) [7, 9]. The Conservation of Resources Model (COR) [8], alternatively, posits that possession of greater resources facilitates higher resilience to stressors and further acquisition of additional resources. Combining these two theories may be mutually beneficial to both. On the one hand, socioecological psychology may contribute to the COR theory as it includes one’s ecological environment (in the current case: a worldwide pandemic, government organizations) and not only one’s close environment as a possible resource. The COR theory, on the other hand, adds to socioecological psychology’s perspective by including different factors shaping one’s thinking, feelings, and behaviors as resources (or lack thereof) that influence how individuals cope with stress. Thus, considering both theoretical perspectives, individual differences in stress reactions may result from a constellation of factors, including sociodemographic factors (i.e., age, gender, religiosity, and employment status), personality factors (i.e., locus of control, resilience), social factors (i.e., loneliness, social support), and socio-political factors (i.e., dimensions of citizens’ trust in government organizations).

### Research context

Socio-ecological psychology, as introduced by Oishi and colleagues [7, 10, 11], offers an objective understanding of human behavior by connecting environmental factors and revealing psychological mechanisms. The COR theory [12] explains how individuals respond to stress by protecting and acquiring resources. Earlier investigations [13] have delved into the psychological stress experienced during the COVID-19 pandemic through the lens of COR theory. They identified that the loss of resources, stress, feelings of loneliness, and concerns were associated with heightened distress and post-traumatic stress, while factors related to resilience filled a protective function. Furthermore, within the realm of public health research, there has been an active push to endorse the utilization and integration of the socioecological model in health interventions to increase individuals’ health [14]. These two theories may complement each other when explaining psychological distress displayed during the COVID-19 pandemic. Socio-ecological psychology helps understand how environmental factors impact individuals’ mental health during a pandemic outbreak, while COR theory provides insights into how different factors shape one’s thinking, feelings, and behaviors as resources (or lack thereof) that affect how individuals cope with stress. Thus, this combined perspective offers valuable insights that are particularly relevant to mental health understanding. By merging these two theories, we offer a broader point of view on individuals’ psychological well-being during a pandemic outbreak.

### The Israeli context

In the unique context of Israel, a country known for its democratic principles and rapidly expanding economy [15], various sociodemographic factors come into play. With a population exceeding nine million, Israel has a large Jewish majority, accounting for roughly 74%, with a noteworthy subgroup of nearly 12% who adhere to ultraorthodox religious beliefs. Most of the remaining population, about 21%, is comprised of Arabs, with the remaining 5% representing individuals from diverse religious and ethnic backgrounds [16]. This sociocultural landscape is marked by a dynamic interplay between traditional collectivist values, deeply rooted in Jewish tradition and socialist ideologies, and a growing trend toward individualism—a shift paralleling developments in many Western countries [17]. This complexity is further shaped by a strong sense of national unity and solidarity, forged in response to historical wars and security threats [17].

Within this multifaceted environment, the Israeli healthcare system stands out for its commitment to universal health insurance (State Health Law, 1994). Regulated by the Ministry of Health, it offers a comprehensive range of healthcare services accessible to all citizens through four health maintenance organizations (sick funds). These organizations provide primary care services through community clinics, while inpatient services are primarily administered by public hospitals, with associated costs covered by the sick funds. Additionally, Israel boasts well-established electronic health records and a robust physical and virtual healthcare infrastructure [18]. Importantly, despite Israel’s remarkable success in the hi-tech industry, socioeconomic disparities persist. This prosperity has not been uniformly distributed, resulting in unequal access to its benefits [15]. However, it is notable that during the COVID-19 pandemic, the Israeli government played a pivotal role in providing a safety net for all citizens. This encompassed dedicated loans for small business owners, rapid and widespread financial assistance, and increased allowances aimed at supporting vulnerable populations, including elderly individuals who faced unemployment during this challenging period.

### Sociodemographic factors

Recent studies relating to COVID-19 [19–22], suggest that *gender* reliably predicts psychological impact: males report less psychological distress than their female counterparts, who evidence moderate levels of anxiety. Accordingly, it was hypothesized that women would report higher levels of psychological distress than would men (H1).

*Age* is an additional factor associated with COVID-19 outcomes, as both the elderly (older than 60) and young adults (aged 18–30) have demonstrated the highest levels of psychological distress, although results vary across studies [19–22]. Notably, a study conducted within Middle Eastern Arab nations revealed that both Generation Y and Generation Z individuals exhibited heightened anxiety in response to extreme-context perceptions, subsequently leading to increased job insecurity due to feelings of alienation, in contrast to Generation X respondents [23]. Moreover, in a study conducted in Israel, older age was associated with lower psychological distress [24], since the Israeli government assured income assistance to those whose wage was negatively affected by COVID-19, and since older individuals were instructed to maintain quarantine more than were young individuals, it was hypothesized that young adults in Israel would report higher levels of psychological distress than would older adults (H2).

Religion has been associated with enhanced ability to handle crises [25]. Religiosity may provide protection against the impact of stressors by facilitating greater coping resources or interpretative frameworks [26]. Therefore, it is critical to investigate and document how religiosity affects individuals’ psychological distress in times of crisis [27]. In line with this notion, religiosity has been found to correlate negatively with distress due to the COVID-19 outbreak [28–30]. Thus, it was hypothesized that religious people would report lower levels of psychological distress than would non-religious people (H3).

Regarding employment status, distress over potential job loss has also been identified as a significant stressor in and of itself [31], particularly during economic crises, previous outbreaks of disease, as well as during the current COVID-19 outbreak [32–35]. In line with these previous studies, it was hypothesized that employed individuals would report lower levels of psychological distress than would unemployed individuals (H4).

### Personal characteristics

Attribution of control is another factor impacting one’s stress reactivity. While some individuals’ *locus of control* is internal and they therefore attribute the outcomes of their life events to their own actions and personal characteristics, others possess an external locus of control, attributing their life events to external forces such as circumstances or luck [36]. In the face of the COVID-19 outbreak, the scope of one’s personal control may be perceived as quite low, since the trajectory of the outbreak is highly contingent upon external forces such as the behavior of others. However, those with an internal locus of control can still be proactive regarding self-protective behaviors such as social distancing, wearing masks, and handwashing, in order to reduce their chances of becoming infected. Indeed, it was found in previous research that individuals with an internal locus of control reported low levels of psychological distress [37] and that external locus of control was associated with symptoms of depression and anxiety [38, 39]. Accordingly, it was hypothesized that an internal locus of control would be negatively associated with psychological distress (H5) and that an external locus of control would be positively associated with psychological distress (H6).

*Resilience* is generally defined as the ability to flexibly adapt to stressful circumstances, which mitigates the impact of negative emotional experiences [40, 41]. Recent research on increasing resilience during the COVID-19 pandemic concluded that it is essential that strategies to promote resilience be developed and implemented to counter psychological distress [42, 43]. Hence, it was hypothesized that higher levels of resilience would be associated with lower levels of psychological distress (H7).

### Social factors

COVID-19 introduced stressors to mental health, including lower levels of *social support* and *loneliness* stemming from social isolation, fear of contracting the disease, economic strain, and uncertainty about the future [44]. Loneliness is a factor widely associated with psychological distress as well as a negative outcome in and of itself [45]. Accordingly, it was hypothesized that higher levels of loneliness would be associated with higher levels of psychological distress (H8).

Social support, a multidimensional construct related to the perception that one’s social network is prepared to assist when necessary, as well as that one can receive concrete resources from others [46], is key to helping one cope with natural disasters [47]. Lack of social support or a decrease in it may increase levels of psychological distress. Indeed, recent research found that individuals with higher psychological distress reported less social support than those with lower psychological distress [48–50]. Accordingly, it was hypothesized that higher levels of social support would be associated with lower levels of psychological distress (H9).

### Socio-political factors

Psychological responses to COVID-19 may also be related to the level of trust in government organizations. Trust is ‘the willingness of a party to be vulnerable to the actions of another party based on the expectation that the other will perform a particular action important to the trustor, irrespective of the ability to monitor or control that other party’ [51, p 712]. Various literature reviews on organizational trust have shown that the dimensions of perceived competence, benevolence, and integrity are recognized as central dimensions in most organizational trust studies [51–53]. These three dimensions are defined as follows for government organizations: perceived competence—the extent to which a citizen perceives a government organization as capable, effective, skillful, and professional; perceived benevolence—the extent to which a citizen perceives a government organization as caring about the welfare of the public and as motivated to act in the public interest; and perceived integrity- the extent to which a citizen perceives a government organization as sincere, truthful, and fulfilling its promises [54, p. 587].

Indeed, a recent study demonstrated that psychological distress was associated with distrust in public health authorities as well as with negative attitudes regarding healthcare professionals [55]. As such, psychological distress due to a pandemic outbreak is not only a stress-induced consequence but results from the interaction between personal and sociopolitical factors. More specifically, since for historical, political, social, and international reasons, Israeli citizens have been experiencing emergency situations for many years and also expect to experience them in the future [56], and since Israelis’ trust in the public sector in general and their evaluation of its performance have always been relatively low [57], it was hypothesized that perceived integrity would not affect Israelis’ psychological distress during the COVID-19 outbreak (H10). Moreover, since the Israeli healthcare system, which handles many aspects of the COVID‐19 crisis, has been relatively effective and highly rated among developed countries [58], it was hypothesized that perceived competence would not affect Israelis’ psychological distress during the COVID-19 outbreak (H11). Nevertheless, as the Government Perceptions Index in Israel has been rising steadily since 2014 [59], and government benevolence in the US was found to be associated with more compliance with government issued guidance [60], it was hypothesized that higher levels of perceived benevolence would be associated with lower levels of psychological distress (H12).

In sum, the complexity of the COVID-19 pandemic experience calls for considerations at many different levels of inquiry: sociodemographic, personality, social, and socio-political. Our aim in this study was to examine individuals’ experiences of the novel coronavirus and their reactions to it as they occurred at the intersection between two perspectives: socio-ecological psychology and the COR theory.

## Methods

### Research population and sample

The research population was comprised of Israeli adults aged 21 and older. For the purpose of the present study, 1,031 subjects were initially sampled. However, to address the problem of missing values, the listwise deletion method was employed [61]. According to this method, in a large enough sample, cases with missing values can be removed from the database without producing biased estimates and without impairing the power of the statistical testing. Hence, 40 respondents who did not fully complete the questionnaire were removed from the statistical analysis, leaving a sample of 991 respondents. Women constituted 52.7% of the sample and men 47.3%, while the mean age was 37.5 years (*SD* = 16.72).

### Sampling method and data collection

The current study was carried out after receiving the approval of the institutional ethics committee for nonclinical research in humans. The questionnaire was distributed online between November 2020 and March 2021, during a COVID-19 outbreak period, across a wide variety of social networks with Hebrew-speaking participants. Before answering the questionnaire, all respondents were asked to read an explanation of the study and provide electronic informed consent. In the explanation, participants were instructed to answer the questionnaire with regard to the COVID-19 outbreak, their experience of it, and their reactions to it. The anonymity of the respondents was ensured by not collecting any identifying details. Contact details of the principal investigator were provided in case the respondents wished to receive further clarifications regarding completion of the questionnaire or participation in the study.

### Research instruments

#### Independent variables

*Locus of control* was assessed by the Internal–External Locus of Control Short Scale (IE-4) designed by Kovaleva [62], which includes two items on an Internal Locus of Control scale and two items on an External Locus of Control scale. The response options ranged from 1 (doesn’t apply at all) to 5 (applies completely). An Internal Locus of Control index was created by calculating the mean of respondents’ answers to two related items: "If I work hard, I will succeed" and "I‘m my own boss". A higher score indicates a higher level of internal locus of control. Also, an External Locus of Control index was created by calculating the mean of respondents’ answers to two related items: "Whether at work or in my private life, what I do is mainly determined by others" and "Fate often gets in the way of my plans". A higher score indicates a higher level of external locus of control. In the present study, the McDonald’s omega (ω) [63] reliability coefficient for Internal Locus of Control items was .684 and .353 for External Locus of Control items.

*Self-reported resilience* was assessed by the Connor-Davidson Resilience Scale [64], which consists of ten statements. On a 5-point Likert scale, respondents were asked to rate to what degree the statements were personally true after encountering a difficult experience (e.g., "Adapt to change", "Deal with whatever comes my way", "See the humorous side of things"). Possible responses ranged from 0 (not true) to 4 (true nearly all of the time). The final resilience score was the sum of answers to all ten items, with higher scores representing higher levels of self-reported resilience. In the present study, Cronbach’s alpha for this scale was .*889*.

*Self-reported loneliness* was examined utilizing the three-item UCLA Loneliness scale [65]. Participants were asked how often they feel that they lack companionship, how often they feel left out, and how often they feel isolated from others. Possible responses were: (1) hardly ever, (2) some of the time, or (3) often. Responses to the three items were totaled, with greater loneliness indicated by higher scores. In the present study, the Cronbach’s alpha for this scale was .*793*.

*Perceived social support* was examined via the Multidimensional Scale of Perceived Social Support (MSPSS) [66]. This instrument is comprised of 12 items that assess the subjective perception of one’s available social support from three different sources: family (e.g., "My family really tries to help me"), significant others (e.g., "There is a special person in my life who cares about my feelings"), and friends (e.g., "My friends really try to help me"). Responses were given on a Likert-type scale from 1 (very strongly disagree) to 7 (very strongly agree). A higher total mean score for all questions indicates higher perceived social support. In the present study, the Cronbach’s alpha for this scale was .*955*.

*Citizen trust in government organizations* [54] was assessed on three dimensions: integrity (e.g., "Government organizations are sincere"), perceived competence (e.g., "The organizations are capable"), and benevolence (e.g., "The organizations act in the best interest of the citizens"), containing three items each. Responses to the items were rated on a five-point Likert scale from 1 (very strongly disagree) to 5 (very strongly agree). The mean score of the relevant items was calculated for each subscale separately, where a higher score indicates that citizens perceive a government organization as competent, benevolent, and integrous to a greater extent. In the present study the internal consistency reliability for the scales (Cronbach’s alpha) was .850 for integrity, .874 for perceived competence, and .930 for benevolence.

Additionally, the following demographic characteristics were collected: gender (female or male), age, employment status (employed or not), and religiosity (religious or not).

#### Dependent variable

*Psychological distress* was assessed using the Kessler Psychological Distress Scale (K6) [67]. Consisting of 6 items, the K6 examines nervousness, hopelessness, irritability, negative affect, fatigue, and worthlessness, experienced over the past 30 days (e.g., "During the last 30 days, about how often did you feel nervous?", "During the last 30 days, about how often did you feel so sad that nothing could cheer you up?", “During the last 30 days, about how often did you feel that everything was an effort?"). Using a five-point Likert scale (revised), items were rated from 0 (absence of the symptom) to 4 (highest level of the symptom). Total scores for the six items ranged from 0 to 24, with higher scores reflecting higher levels of psychological distress. The internal consistency reliability (Cronbach’s alpha) was .852 for the present study.

There is no consensus among the researchers as to cutoff criteria that allow discernment of serious mental illness cases. For example, Kessler et al. [67], the K6 developers who clinically validated the K6, suggested that based on diagnostic criteria of the DSM-IV, the K6 cutoff point of 13 allows discernment of serious mental illness cases in population-based surveys [68, 69]. Cornelius et al. [70] and Staples et al. [71] utilized in their studies an optimal cutoff point of 14, while Arnaud et al. [72] and Lace et al. [73] reported a cutoff score of 10 for the K6. In the current study only 7.5% of the participants received a score ≥ 13, implying that a sufficient proportion of them did not experience a severe level of mental distress [67, 69, 74]. A cutoff point of ten would imply that 17.0% of the respondents have a severe level of mental distress [72, 73], while a cutoff point of 14 would imply that 3.7% of the respondents have a severe level of mental distress [70, 71].

#### Statistical analyses

Descriptive measures (means, standard deviations, or percentages) were obtained for all research variables (see Table 1). A hierarchical regression analysis was conducted to examine the association between a series of independent variables and psychological distress among adults during a COVID-19 outbreak in Israel (see Table 2). The maximal VIF measure of predictors was 3.32, indicating no problem of multicollinearity in the regression model (for correlations between the research variables see Table 3). The number of independent variables entered in the regression was also adequate for the sample size (*n* = 991) [75].

**Table 1:** Descriptive statistics of the research variables (n = 991).

Variables	Statistics			
	N	%	Mean	SD
Age			37.5	16.72
Gender				
Male	469	47.3%		
Female	522	52.7%		
Religiosity				
Religious	465	46.9%		
Not religious	526	53.1%		
Employment				
Employed	648	65.4%		
Not Employed	343	34.6%		
Internal locus of control ^a^			3.66	.89
External locus of control ^b^			2.33	.78
Resilience ^c^			27.36	7.35
Loneliness ^d^			4.8	1.55
Social support ^e^			5.75	1.17
Competence ^f^			2.67	.95
Benevolence ^g^			2.74	.99
Integrity ^h^			2.22	.95
Psychological distress ^i^			5.68	4.17

Notes.

a. Short Scale for the Assessment of Locus of Control (IE-4). Scores on this item range from 1 to 5, with higher scores indicating an internal locus of control.

b. Short Scale for the Assessment of Locus of Control (IE-4). Scores on this item range from 1 to 5, with higher scores indicating an external locus of control.

c. Connor-Davidson Resilience Scale. Scores on this item range from 0 to 40, with higher scores indicating greater levels of resilience.

d. UCLA Loneliness scale. Scores on this item range from 3 to 9, with higher scores indicating greater levels of loneliness.

e. Multidimensional Scale of Perceived Social Support (MSPSS). Scores on this item range from 1 to 7, with higher scores indicating greater levels of perceived social support.

f. Citizen trust in government organizations scale. Scores on this item range from 1 to 5, with higher scores indicating a greater extent to which a citizen perceives government organizations as competent.

g. Citizen trust in government organizations scale. Scores on this item range from 1 to 5, with higher scores indicating a greater extent to which a citizen perceives government organizations as benevolent.

h. Citizen trust in government organizations scale. Scores on this item range from 1 to 5, with higher scores indicating a greater extent to which a citizen perceives government organizations as integrous.

i. Kessler Psychological Distress Scale (K6). Scores on this item range from 0 to 24, with higher scores indicating a greater level of psychological distress.

**Table 2:** Summary of the hierarchical regression analysis for variables explaining psychological distress among Israeli adults during the Covid-19 pandemic.

	B	Std. Error	Beta	p	Adj.R^2^	ΔR^2^	F
Step 1					.022	.026	F_(4,987)_ = 6.503***
Age	-.016	.008	-.065	.038			
Gender	.584	.265	.070	.028			
Religiosity	.968	.263	.116	.000			
Employment	.420	.279	.048	.132			
Step 2					.139	.120	F_(7,984)_ = 23.88***
Age	-.006	.007	-.026	.387			
Gender	.609	.252	.073	.016			
Religiosity	1.072	.247	.128	.000			
Employment	.624	.262	.071	.017			
Internal Locus of Control	-.685	.142	-.146	.000			
External Locus of Control	1.205	.163	.225	.000			
Resilience	-.090	.017	-.158	.000			
Step 3					.289	.150	F_(9,982)_ = 45.61***
Age	-.009	.007	-.034	.207			
Gender	.623	.234	.075	.008			
Religiosity	.872	.226	.104	.000			
Employment	.464	.239	.053	.052			
Internal Locus of Control	-.436	.131	-.093	.000			
External Locus of Control	.993	.149	.186	.000			
Resilience	-.057	.016	-.100	.000			
Loneliness	.752	.076	.279	.000			
Social support	-.815	.104	-.229	.000			
Step 4					.299	.013	F_(12,979)_ = 36.19***
Age	-.007	.007	-.029	.293			
Gender	.604	.234	.072	.010			
Religiosity	.571	.236	.068	.015			
Employment	.536	.238	.061	.025			
Internal Locus of Control	-.398	.130	-.085	.002			
External Locus of Control	1.024	.148	.191	.000			
Resilience	-.050	.016	-.089	.002			
Loneliness	.730	.075	.271	.000			
Social support	-.806	.104	-.227	.000			
Competence	-.043	.203	-.010	.832			
Benevolence	-.435	.204	-.104	.033			
Integrity	-.044	.212	-.010	.834			

***p< .001

Note. Gender (dummy): 0-male, 1- female; Religiosity (dummy): 0- religious, 1- not religious; Employment (dummy): 0- employed, 1- not employed

**Table 3:** Correlations between the research variables.

Variables	1	2	3	4	5	6	7	8	9	10	11
1. Age	-										
2. Gender	-.006										
3. Religiosity	-.016	--									
4. Employment	-.007	--	--								
5. Internal locus of control	.083**	-.070*	.039	.024							
6. External locus of control	-.137***	-.107***	.003	-.060	-.147***						
7. Resilience	-.024	-.050	.039	.039	.177***	-.097**					
8. Loneliness	.000	.121***	.072*	.084**	-.092**	.071*	-.052				
9. Social support	.002	.134***	.005	.053	.181***	-.136***	.243***	-.230***			
10. Competence	.065*	.005	-.280***	.040	.093**	.008	.091**	-.090**	.026		
11. Benevolence	.033	-.028	-.295***	.077*	.077*	.015	.096**	-.096**	.067*	.771***	
12. Integrity	.144***	-.062	-.274***	.019	.081*	-.007	.084**	-.092**	.041	.763***	.787***

Note.

*p < .05

**p < .01

***p< .001

Gender (dummy): 0-male, 1-female; Religiosity (dummy): 0-religious, 1-not religious; Employment (dummy): 0-employed, 1-not employed.

Positive significant correlations of the dummy variables (2–4) with the continuous variables (1, 5–12) imply that women scored higher than men, not religious persons scored higher than religious, and not employed scored higher than employed in those variables. Accordingly, negative significant correlations of the dummy variables (2–4) with the continuous variables (1, 5–12) imply that men scored higher than women, religious persons scored higher than not religious, and employed scored higher than not employed in those variables.

## Findings

Age, gender, religiosity, and employment status were entered in the first step of the regression model to control for the demographic variables (*F*_(4,987)_ = 6.503, *p* = .000). Internal and external locus of control and resilience were entered in the second step (*F*_(7,984)_ = 23.88, *p* = .000) and loneliness and social support were entered in the third step (*F*_(9,982)_ = 45.61, *p* = .000). The three components of trust in government organizations (competence, benevolence, and integrity) were entered in the fourth step (*F*_(12,979)_ = 36.19, *p* = .000).

Including all independent variables in the last (fourth) step of the regression model revealed that, as hypothesized, women (H1), non-religious people (H3), and the unemployed (H4) reported higher levels of psychological distress than men (*β* = .072, *p* = .010), religious people (*β* = .068, *p* = .015), and those who were employed (*β* = .061, *p* = .025). An internal locus of control (H5) (*β* = -.085, *p* = .002), resilience (H7) (*β* = -.089, *p* = .002), social support (H9) (*β* = -.226, *p* < .001), and benevolence (H12) (*β* = -.104, *p* = .003) were negatively associated with psychological distress. In contrast, self-reported loneliness (H8) (*β* = .271, p = .000) and external locus of control (H6) (*β* = .191, *p* = .000) positively predicted the level of respondents’ psychological distress. However, no association was detected between respondents`age (H2) (*p* = .293), the extent to which they perceive the government as competent (H11) (*p* = .832) and integrous (H10) (*p* = .834), and psychological distress. Together, the independent variables accounted for 29.9% of the variance in psychological distress in the current sample.

## Discussion

The aim of this research was to examine individuals’ experiences of COVID-19 and reactions to it from the perspectives of socio-ecological psychology [7, 9] and the COR theory [8], thus proposing an integrative outlook which is highly suitable for mental health care practitioners. A combination of the two theories suggests that individuals’ psychological functioning and use of resources may affect their broader social habitats, which in turn may shape their mind, behavior, and use of resources. Accordingly, it is possible to view COVID-19 as a real-life phenomenon that relates to the broader social and natural ecology. Moreover, COVID-19 may be seen as a “stressful situation”, using Hobfoll’s [12] definition of stress that accommodates both the socio-ecological psychological and COR theory perspectives: stress is “a reaction to the environment in which there is (a) the threat of a net loss of resources, (b) the net loss of resources, or (c) a lack of resource gain following the investment of resources” (p. 516).

With this notion in mind, we suggest viewing age, gender, religiosity, and employment status as condition resources, meaning resources to the extent they are valued and sought after [12]. Our first research finding indicates that, as hypothesized, women and not religious and unemployed individuals reported higher levels of psychological distress than men, religious people, and those who were employed. These findings may be understood as psychological distress caused by lack of resources. Regarding gender, especially in Israel, a highly familial country in which women are still perceived as the primary caretaker for childrearing and providing for their needs [77], prior to COVID-19 women were already managing with lesser resources of various kinds compared to men, particularly time, money, and career opportunities. Hence, COVID-19 caused a disproportionate loss of resources among women compared to men [78], thereby causing more psychological distress. This finding is also supported by previous research suggesting that females are more significantly affected by psychological distress than their male counterparts [19–22].

Religiosity may also serve as a resource that provides a buffer against stress by providing an interpretative framework, enhancing coping resources, or facilitating access to social support [26]. Thus, and as found in previous research, religious people feel less psychological distress resulting from the COVID-19 outbreak [28]. Moreover, stress may occur as a result of the actual loss of resources [12]. Thus, unemployment may be viewed as a loss of resources that causes stress and psychological distress; accordingly, it was found in previous research that distress over potential unemployment was a significant stressor during the COVID-19 outbreak [32–34]. Regarding age, in contrast to that hypothesized, the research findings detected no association between respondents’ age and psychological distress. These findings may be understood in light of previous research indicating that adults who were older typically displayed lower stress reactivity and more effective emotional regulation and, in light of often having more experience of being alone and managing life-threatening medical situations, they also had more personal resources and were less psychologically distressed due to the COVID-19 outbreak [78–80].

Resilience and internal/external locus of control may be seen as resources in one’s personality characteristics, meaning personal traits and skills that aid in resisting stress, such as having a positive sense of self and believing one has control over their circumstances [12]. Indeed, our research findings indicated that internal locus of control and resilience were negatively associated with psychological distress, while external locus of control was found to be positively associated with it. These findings are in line with previous research indicating that during COVID-19 individuals with an internal locus of control reported lower levels of psychological distress [37] and that resilience may counter psychological distress [42, 43]. At the same time, the finding regarding the external locus of control is also consistent with other findings on the association between external locus of control and symptoms of depression and anxiety [38].

From the point of view of socio-ecological psychology [7], loneliness and social support are important social factors, which from the COR perspective can be defined as resources whose presence or absence can increase or decrease the individual’s ability to deal with stress and crises. Indeed, the current research findings indicate that social support was negatively associated with psychological distress while self-reported loneliness positively predicted psychological distress levels. These findings are in line with previous research indicating that individuals with higher psychological distress reported fewer resources in social support than those with lower psychological distress [48–50]. Moreover, our findings are also in line with previous research linking self-reported loneliness with a range of deleterious physiological and psychiatric outcomes at all times as well as during the COVID-19 pandemic [81, 82].

Regarding dimensions of citizens’ trust in government organizations, our findings indicate that perceived benevolence was negatively associated with psychological distress. However, no association was detected between perceived competence and perceived integrity and psychological distress. These findings emphasize the importance of the benevolent image of government organizations in the eyes of the public, a perception that in fact acts as a protective factor which reduces psychological distress during acute crises [59]. Hence, perceiving government organizations as benevolent may also be viewed by the COR theory as an “energy resource” that provides the ability to attain other resources. Through such trust, individuals may obtain resources they lack, such as social capital, knowledge, and a sense of inclusion which contribute to reduction of the psychological distress. Perceiving government organizations as benevolent may also be viewed as an energy resource for governments themselves, as trusting citizens enable governments to function more smoothly in stressful times.

In sum, this research was an attempt to further the understanding of the origin, maintenance, and regional distribution of social habitats and their relationships with the human mind and behavior [7]. The results indicate that individuals with greater resources were less likely to suffer from psychological distress during the COVID-19 outbreak. It was not a single resource or a specific stress that caused psychological distress, rather the integration between the broader social habitats and the human mind, behavior, and resources.

That said, several limitations to our research should be considered. First, our study was conducted in Israel, a developed Western nation recognized for its multicultural population on one hand, and for its distinctive close-knit community dynamics, common in relatively smaller countries, on the other [15, 83]. Consequently, it is essential to recognize that the experience of globalization varies significantly between countries and even within different regions of the same country, occurring at differing paces and scales [84]. Therefore, it is imperative to assess the applicability and relevance of our findings in diverse international contexts. Moreover, this study was based on self-reports and a cross-sectional design was utilized to collect data. Accordingly, no information was obtained on psychological distress participants may have experienced during previous periods. Further, as data was collected through an online survey, there is the possibility of a response bias, so future studies may choose to utilize alternative methods of data collection. Also, the reliability of the External Locus of Control scale in our study is significantly lower than in previous studies, such as in that conducted by Kovaleva et al. (ranging from .53 to .63) [85] and in the study conducted by Nießen et al. (ranging from .63 to .69) [86]. In future studies this should be taken into account and the sensitivity of this scale to intercultural differences should be reexamined. Beyond that, the current study examined the perception of the individual’s religious identity as a dichotomous variable: whether the person defines himself as religious or not. It is recommended that future studies examine religious self-identity as a continuum and also address the aspect of the individual’s belonging to a certain faith.

### Practical implications

The interpretation of the current study results through the lens of the theoretical frameworks of socio-ecological psychology and the COR theory produces several important implications for practice. The ongoing global battle against the COVID-19 pandemic has wrought profound changes in personal, social, and economic domains, challenging individuals and societies worldwide to adapt and navigate unprecedented challenges. Within this context, it is essential to acknowledge that individuals in Israel, as well as in similar regions, often possess a heightened perception of susceptibility to ambiguous or uncertain events. This perception has led to the formulation of deeply ingrained beliefs and institutions designed to shield against these uncertainties [23]. In light of this, policymakers must direct their efforts toward initiatives that extend beyond addressing immediate pandemic-related challenges and instead promote long-term resilience within society. This may involve investments in mental health support programs. As our findings demonstrate how psychological distress may result from the integration between the individual’s broader social habitats and their mind, behavior, and resources, mental health professionals may help clients achieve a reflective critique on their wide environments and encourage them to be more socially active. This may be done through therapy focusing on one’s locus of control (especially internal) and resilience, and through group therapy, which may reduce loneliness and encourage social support. Additionally, during a pandemic, when we are surrounded by the “politics of pandemics,” there is a need for principled activism based on values such as social justice and human rights that counters both the excesses of neoliberalism and provides alternatives to populist notions of dystopia. Moreover, policymakers must pay particular attention to the effects of COVID-19 on women and unemployed individuals (whether prior to, during, or post-COVID-19). Within this context, it is evident that the development of a competitive national innovation and entrepreneurial ecosystem, as underscored by Berman et al. [84, 87, 88], takes on heightened significance for Israel’s economic progress [15]. Such an ecosystem is not merely a conduit for economic growth; it is also an essential element in fortifying societal resilience against the disruptions wrought by crises such as the pandemic. Policymakers must also take steps to increase trust in government organizations (in particular their benevolence) among these specific subpopulations as well as among the general population, because this works as a protective factor that helps the population reduce psychological distress in times of acute crisis. A trustworthy government organization may be viewed as a “wide social habitat” that shapes minds and behaviors; indeed, individuals’ psychological functioning and interactions with proximal contexts may affect the functioning and trustworthiness of government organizations. Thus, it is important that policymakers at all levels strengthen bonds of trust through a transparent dialogue between citizens and government.
